# Auditory hallucinations, childhood sexual abuse, and limbic gray matter volume in a transdiagnostic sample of people with psychosis

**DOI:** 10.1038/s41537-022-00323-y

**Published:** 2022-12-30

**Authors:** Zachary B. Millman, Melissa Hwang, Valerie J. Sydnor, Benjamin E. Reid, Joshua E. Goldenberg, Jessica N. Talero, Sylvain Bouix, Martha E. Shenton, Dost Öngür, Ann K. Shinn

**Affiliations:** 1 Psychotic Disorders Division, McLean Hospital, Belmont, MA USA; 2 Department of Psychiatry, Harvard Medical School, Boston, MA USA; 3 Psychiatry Neuroimaging Laboratory, Brigham and Women’s Hospital, Boston, MA USA

**Keywords:** Biomarkers, Psychosis

## Abstract

Childhood sexual abuse (CSA) is a potentially unique risk factor for auditory hallucinations (AH), but few studies have examined the moderating effects of sex or the association of CSA with limbic gray matter volume (GMV) in transdiagnostic samples of people with psychotic disorders. Here we found that people with psychotic disorders reported higher levels of all surveyed maltreatment types (e.g., physical abuse) than healthy controls, but people with psychotic disorders with AH (*n* = 41) reported greater CSA compared to both those without AH (*n* = 37; *t* = −2.21, *p* = .03) and controls (*n* = 37; *t* = −3.90, *p* < .001). Among people with psychosis, elevated CSA was most pronounced among females with AH (sex × AH status: *F* = 4.91, *p* = .009), held controlling for diagnosis, medications, and other maltreatment (*F* = 3.88, *p* = .02), and correlated with the current severity of AH (*r* = .26, *p* = .03) but not other symptoms (*p*’s > .16). Greater CSA among patients related to larger GMV of the left amygdala accounting for AH status, diagnosis, medications, and other maltreatment (*t* = 2.12, *p* = .04). Among people with psychosis, females with AH may represent a unique subgroup with greater CSA. Prospective high-risk studies integrating multiple measures of maltreatment and brain structure/function may help elucidate the mechanisms linking CSA with amygdala alterations and AH.

## Introduction

Psychotic disorders are highly heterogeneous in underlying neurobiology, risk factors, symptom presentation, and functional trajectory. Such variability challenges efforts to understand the pathophysiology of these disorders and undermines the utility of current diagnostic paradigms which imply discreteness across clinical entities. Interest has grown in identifying more clinically and biologically homogenous patient subgroups to aid in treatment development, clinical management, and early detection. The study of auditory hallucinations (AH) has been a promising avenue in this respect. AH are endorsed by an estimated 76% of people with schizophrenia, 72% of schizoaffective disorder, and 34% of psychotic bipolar disorder^1^, and are also seen in nonpsychotic disorders^2^ and in the general population^3^. Importantly, clinically significant AH are often distressing and are associated with greater functional difficulties, increased risk of relapse, and greater risk of suicide among people with psychosis^4,5^. Yet, AH are treatment refractory in 25-30% of cases^6^, suggesting a greater need to understand the nature of AH in psychotic disorders.

A relatively well-studied risk factor for AH is childhood maltreatment. Exposure to maltreatment in childhood increases the risk of AH, and psychosis more generally, in early adulthood^7^. Although some studies suggest that different types of maltreatment do not differentially increase risk for psychosis^8^, other evidence suggests that exposure to childhood sexual abuse (CSA) may be an especially potent predictor of AH^9^. This pronounced association between CSA and AH has been observed in studies spanning a range of populations, including adults with psychotic disorders^10^, young people at clinical high risk for psychosis^11^, non-psychotic voice-hearers^12^, and the general population^13^.

Importantly, females are at greater risk of CSA victimization than males. In the United States, prevalence rates for rape are 18.3% for females and 1.4% for males^14^. Among psychiatric inpatients, 48% of females and 28% of males endorsed CSA^9^, mirroring the sex imbalance seen in the general population. Considering the higher risk of CSA in females together with the increased risk of AH among individuals who have experienced CSA, female victims of CSA may be at particularly elevated risk for developing AH later in life.

Exposure to maltreatment including CSA can have neurodevelopmental consequences on brain structure and function^15^. Among other regions including the orbitofrontal and dorsolateral prefrontal cortices, meta-analyses of whole-brain studies find that the amygdala and hippocampus — which are key regions of the limbic system important for the processing of emotions and episodic memory, respectively — are among the structures most commonly implicated in studies of maltreatment^16–18^. These limbic regions have high concentrations of stress hormone receptors and are thus particularly susceptible to the cascade of physiological and neuroendocrine events, including downstream effects on neuronal remodeling (e.g., changes in dendritic structure, synaptic density, etc.), that are mediated by the hypothalamic-pituitary-adrenal axis and set in motion by childhood maltreatment and other forms of toxic stress^19^. Although findings of hippocampal volume reduction are fairly consistent^20^, studies are mixed with respect to the direction of amygdala volume alteration following maltreatment or other types of adversity, with studies showing both lower^21–24^ and higher^25–27^ amygdala volumes. Some potential sources of this between-study variability may include study population^15^, co-occurrence of multiple maltreatment dimensions^28^, and medication effects on brain volume^29^, suggesting the importance of addressing these considerations in study design and analyses.

Importantly, some of the neural substrates of AH overlap with those associated with maltreatment exposure. Several theories on the pathophysiology of AH highlight the roles of disrupted memory, learning, and emotion systems, implicating the involvement of the hippocampus, amygdala, and other limbic structures^30,31^. For example, abnormal structure or function of these areas could lead to memory storage or retrieval impairments and, subsequently, aberrant mental associations or unintended activation of memories – experienced phenomenologically as AH^30^. Studies of people with psychosis have reported altered limbic activity during the experience of AH^32,33^, during emotion processing^34^ and in response to AH-like stimuli^35^. Evidence also points to aberrant functional connectivity between limbic and cortical language areas^36^, suggesting that potentially stress-sensitive memory and emotion networks may act in conjunction with language network disruptions to bring about AH.

While many studies have used functional magnetic resonance imaging to examine regional activity or connectivity associated with AH, it is difficult to disentangle whether such findings reflect a trait or state abnormality associated with AH. In contrast, analyses of brain structure, such as gray matter volume (GMV), can provide insights about trait-level structural abnormalities associated with AH, including those which may be a consequence of childhood exposure to maltreatment. A better understanding of the structural correlates of maltreatment among people with AH would complement existing functional neuroimaging studies and support a more integrated model of AH. Although no studies to date have longitudinally examined brain structure in individuals with childhood maltreatment who prospectively develop AH, some (though not all) structural MRI studies have found volume alterations in limbic regions in association with AH^37–42^. The overlap between structural brain substrates associated with AH and childhood maltreatment suggests a potential pathway by which CSA could increase risk for AH.

Here we sought to examine the interrelations between CSA, AH, sex, and GMV of the amygdala and hippocampus among people with psychotic disorders. Our first aim was to determine whether people with psychotic disorders and lifetime AH report different levels of CSA history than people with psychotic disorders and no lifetime AH (NAH), and whether this effect is moderated by sex. Our second aim was to determine whether CSA is associated with GMV of the hippocampus and amygdala. We hypothesized that people with psychosis and AH would endorse greater CSA history relative to people with psychosis and NAH and healthy controls, even after accounting for other maltreatment types (e.g., physical abuse, neglect), DSM diagnostic class, and antipsychotic medication. We expected that this effect would be moderated by sex such that the positive gradient of CSA severity (from control to NAH to AH) would be strongest among females. Finally, we hypothesized that greater CSA among patients would be associated with greater GMV reductions in both the hippocampus and amygdala, even when accounting for other types of maltreatment, sex, AH status, DSM diagnostic class, and antipsychotic medication.

## Results

We collected structural MRI data from 118 participants. Six were missing GMV data due to poor scan quality, yielding 112 participants with usable GMV data. There were no age, sex, or diagnostic (AH status or Diagnostic and Statistical Manual of Mental Disorders [DSM] diagnosis, coded as schizophrenia spectrum vs. bipolar disorder) differences between those with versus without missing data. To maximize power, participants were excluded pairwise per analysis (i.e., participants were not excluded from all analyses if they lacked data on one variable). The three groups were clinically and demographically well matched with the following exceptions: both patient groups had completed less education than healthy controls, and AH had higher mean chlorpromazine (CPZ) equivalents than NAH (Table 1). All variables met basic assumptions for the analyses planned.

**Table 1:** Demographic and clinical characteristics of the sample.

Variable	AH (*n* = 41)	NAH (*n* = 37)	HC (*n* = 37)		
	M (*SD*) or *N* (%)			*F*/*t*/χ^2^	*p*
Age	33.67 (*7.22*)	29.73 (*7.33*)	32.43 (*7.98*)	2.75	0.07
Female	21 (51.2)	16 (43.2)	20 (*54.1*)	1.11	0.57
Education (years)^a^	15.24 (*2.40*)	15.19 (*2.18*)	17.22 (*2.37*)	9.25	<0.001
Race^b^				0.08	0.96
White	26 (*70.3*)	27 (*73*)	29 (*70.7*)		
Black	3 (*8.1*)	6 (*16.2*)	6 (*14.6*)		
Asian	6 (*16.2*)	4 (*10.8*)	4 (*9.8*)		
>1 race	2 (*5.4*)	0 (*0*)	2 (*4.9*)		
CPZ equivalents	341.01 (*398.50*)	192.97 (*197.37*)	–	−2.0	0.05
Total medication load^c^	3.15 (*2.11*)	2.77 (*2.03*)	–	−0.78	0.44
Diagnosis					
Bipolar I	16 (38.1)	19 (52.8)		1.38	0.24
SZ/SZA	26 (61.9)	17 (47.2)		1.69	0.19
Current symptom severity					
PSYRATS-AH	12.68 (*14.48*)	0.0 (*0.0*)	–	−5.61	<0.001
SAPS^d^	15.79 (*7.24*)	11.5 (*5.11*)	–	−3.02	0.003
SANS	13.02 (*5.12*)	11.72 (*5.72*)	–	−1.06	0.30
YMRS	11.83 (*9.15*)	8.56 (*7.58*)	–	−1.70	0.09
MADRS	13.07 (*10.29*)	10.20 (*10.33*)	–	−1.22	0.23

*AH* patients with lifetime auditory hallucinations, *NAH* patients without lifetime auditory hallucinations, *HC* healthy controls, *CPZ* chlorpromazine, *SZ* schizophrenia, *SZA* schizoaffective disorder, *PSYRATS*-*AH* Psychotic Symptom Rating Scale, Auditory Hallucinations Subscale, *SAPS* Scale for the Assessment of Positive Symptoms, *SANS* Scale for the Assessment of Negative Symptoms, *YMRS* Young Mania Rating Scale, *MADRS* Montgomery-Asberg Depression Rating Scale.

^a^Both patient groups endorsed fewer years of education than controls. Patient groups did not differ from one another.

^b^Due to small cell sizes, for statistical comparison of race distributions across AH status, these data were regrouped into “White” and “non-White” to satisfy the requirement that chi square cells include at least 5 observations.

^c^Total medication load calculated using the Phillips method^94^.

^d^Values in this table reflect total symptom scores; however, in primary analyses involving the SAPS, we excluded the three auditory hallucinations items from the total SAPS score.

**Fig. 1:**
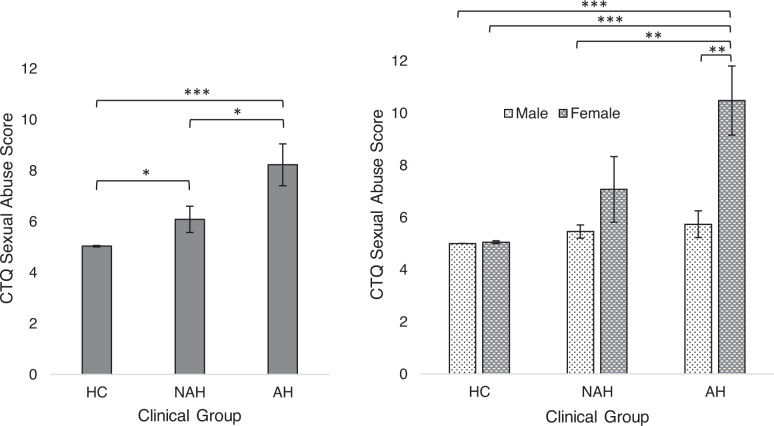
Childhood sexual abuse exposure across clinical group and sex. Differences in exposure severity between (**A**) AH, NAH, and HC groups and between (**B**) AH, NAH, and HC groups by participant sex. Error bars represent standard deviations. AH, psychotic disorder with auditory hallucinations; CTQ childhood trauma questionnaire, HC healthy control, NAH psychotic disorder with no auditory hallucinations. *p < .05, **p < .01, **p < .001.

Table 2 displays results of analyses of variance (ANOVAs) comparing the three groups on the five Childhood Trauma Questionnaire (CTQ) domains. Both AH and NAH endorsed significantly greater levels of all trauma types than healthy controls. In the case of CSA, however, the two patient groups differed from one another, with AH reporting significantly greater histories of CSA than NAH (Fig. 1a). Consistent with our hypotheses, a 2×2 ANOVA with AH status and sex as factors revealed that this gradient effect for CSA was driven by female patients with AH, who reported the highest levels of CSA across clinical groups and sex (sex × AH status interaction: *F* [2, 110]=4.91, *p* = 0.009; Fig. 1b). An analysis of covariance (ANCOVA) indicated that the relation between CSA and AH status (among patients) was robust to controlling for sex, DSM diagnostic class, CPZ equivalents, and all other CTQ maltreatment types (*F* [2, 106] = 3.88, *p* = 0.02; Table S1). No significant differences in the CTQ minimization/denial index were observed across sex or AH status (Table S2**)**.

**Table 2:** Means, standard deviations, and statistical comparisons of maltreatment histories across healthy controls and patients with or without lifetime auditory hallucinations.

CTQ Dimension	AH (*n* = 40)	NAH (*n* = 36)	HC (*n* = 35)		AH vs. HC	NAH vs. HC	AH vs. NAH
	M (*SD*)			*F*	*t*		
Sexual abuse	8.23 (*5.19*)	6.08 (*3.11*)	5.03 (*0.17*)	7.82**	−3.9***	−2.03*	−2.21*
Physical abuse	7.5 (*4.13*)	7.13 (*2.86*)	5.51 (*1.04*)	4.45*	−2.94***	−3.12**	−0.44
Emotional abuse	8.90 (*4.61*)	8.58 (*4.43*)	5.17 (*2.54*)	9.61*	−4.25***	−4.0***	−0.31
Emotional neglect	10.58 (*4.71*)	10.83 (*5.07*)	6.66 (*2.31*)	10.89***	−4.66***	−4.48***	0.23
Physical neglect	7.43 (*3.56*)	7.25 (*2.69*)	5.20 (*0.53*)	7.90**	−3.91***	−4.49***	−0.24

*F* statistic represents results of analyses of variance, *t* statistics represent post-hoc comparisons. *CTQ* Childhood Trauma Questionnaire, *AH* patients with auditory hallucinations, *NAH* patients without auditory hallucinations, *HC* healthy control.

*p < 0.05, **p < 0.01, ***p < 0.001.

In patients, CSA scores and AH severity were significantly positively correlated (*r* = 0.26, *p* = 0.03). Specificity analyses suggested that CSA was not correlated with overall positive (excluding AH items; *r* = 0.17, *p* = 0.16), negative (*r* = −0.01, *p* = 0.97), manic (*r* = 0.16, *p* = 0.17), or depressive symptom severity (*r* = 0.06, *p* = 0.63). Results of a linear regression suggested no sex × CSA interaction in predicting AH severity despite a statistical trend (Table S3), suggesting a potentially similar effect of CSA on AH severity across males and females; however, CSA remained significantly positively associated with AH severity when controlling for sex, DSM diagnostic class, CPZ equivalents, and other maltreatment dimensions (*b* = 0.77 [0.32], *t* [69]=2.41, *p* = 0.02; Table S4), suggesting the relation between CSA and AH severity was not explained by patient diagnosis or co-occurrence of other maltreatment experiences.

Having established the presence of a unique relation between CSA and AH, we sought to relate limbic GMV with CSA in patients. Higher CSA scores were significantly positively correlated with GMV of the left amygdala (*r* = 0.30, *p* = 0.01), but not the right amygdala (*r* = 0.12, *p* = 0.33) or the left (*r* = 0.06, *p* = 0.65) or right hippocampus (*r* = 0.18, *p* = 0.14). Linear regressions revealed no significant sex × CSA interaction in predicting left amygdala GMV (Table S5), suggesting a similar effect of CSA on GMV across males and females. The relation between CSA and GMV was robust to controlling for AH status, DSM diagnostic class, CPZ equivalents, and all other maltreatment types (*b* = 1.69 ×10^−5^ [.00], *t* [71]=2.52, *p* = 0.01; Table S6), suggesting the CSA-amygdala GMV relation was not explained by these covariates.

## Discussion

This study examined the interrelations of CSA, amygdala and hippocampal GMV, sex, and AH in a transdiagnostic sample of adults with psychotic disorders. Consistent with our hypotheses, we found that (1) patients with lifetime AH, compared to those with NAH or healthy controls, reported more severe histories of CSA, and that patients with more severe current AH symptoms reported more severe CSA histories; (2) females with lifetime AH reported the most severe CSA histories; and (3) CSA severity was associated with GMV of the amygdala, although contrary to expectations individuals with higher CSA severity showed greater (not lower) GMV, a relation that held when considering a range of covariates including other maltreatment types. No association was observed between CSA and hippocampal GMV. These findings have implications for our understanding of potential pathways by which distinct clinical presentations of psychosis may develop.

While maltreatment histories in participants with psychotic disorders were greater relative to healthy controls across all five domains of childhood maltreatment, reports of CSA (and only CSA) were especially severe among those with AH, and the most pronounced CSA-AH relations were among female participants. Supplementary analyses suggested these findings were not accounted for by different CSA histories across DSM diagnostic class or by CPZ equivalents or other co-reported maltreatment types. A growing number of studies has demonstrated specific associations between CSA and AH^9,10,12,43^. Although our cross-sectional study is unable to determine a causal relation between the two, when considered alongside the consistent sex differences observed in maltreatment epidemiology^14^, these findings may suggest that female children could be at risk for later AH due to higher exposures to CSA victimization. To test this hypothesis further, it will be important to determine whether males and females are equally susceptible to AH once exposed or if there are sex- or gender-specific effects; longitudinal studies of youth at risk for psychosis will be valuable in addressing this question. Although most individuals exposed to CSA do not develop psychosis^44^, our findings align with the literature in suggesting that additional attention should be paid to the association between CSA and AH among help-seeking individuals with emerging psychopathology. In particular, women with psychosis often face unique (culturally mediated) challenges throughout recovery^45^, and maltreatment histories in psychotic disorders have been identified as important considerations for the development of relapse prevention plans^46^. Treatments that address the intersection between maltreatment, female sex, and AH could thus form the basis of personalized interventions for some individuals.

An important goal of this work was to link a specific environmental risk factor with structural differences in key limbic brain regions among individuals with psychotic disorders. We observed that greater CSA severity was associated with larger GMV of the left amygdala in participants with psychosis, even when accounting for other maltreatment types, AH status, DSM diagnostic class, and CPZ equivalents. Confidence in these results is further strengthened by the observation that psychotic illness and antipsychotic medication are generally both associated with thinning and contraction of the amygdala^47–49^, rather than enlargement as seen here. Notably, no other maltreatment type was a significant predictor of amygdala volume in our models, potentially suggesting some specificity to the CSA-amygdala GMV relation in this sample. It has been suggested that trauma exposure during sensitive developmental stages results in lasting structural alterations and abnormal communication between the amygdala and other regions or networks important for learning, memory, and emotion/salience processing, such as the anterior cingulate and insular cortices^50,51^. Further, “betrayal trauma” events – those characterized by violations of interpersonal trust between the perpetrator and victim, such as CSA – have been associated with altered activation of these regions during a trust violation task^52^. Altered amygdala structure^53,54^, function^34^, and connectivity^55^ have also been reported in relation to hallucinations. For those with a preexisting vulnerability to psychosis, it is possible that the neurodevelopmental consequences of CSA may lead to abnormal “escape” of emotional memory or formed associations^30^, including sensory experiences previously encoded during trauma, potentially leading to AH. More research is needed to understand the complex psychological, perceptual, and neurodevelopmental mechanisms contributing to the association between distinct features of childhood maltreatment and psychopathology.

While some researchers have found that early maltreatment is associated with lower amygdala volume^56^, our finding of larger amygdala volume among those with more severe CSA histories is consistent with several prior reports investigating the effects of early maltreatment on amygdala volume^25–27^ and is also supported by animal models. Although translation of animal findings to humans should be made with caution, seminal studies of rats have shown that pyramidal-like neurons in the basolateral amygdala, a subarea of the amygdala important for aversive learning^57^, show hypertrophy of dendritic structures following chronic stress^58,59^. Dendritic hypertrophy is consistent with amygdalar enlargement^19^. If these findings can be applied to humans, they suggest that maltreatment-related amygdala volume increases such as those seen presently could be driven primarily by the basolateral subregion, which could represent a potential treatment target. Unfortunately, our FreeSurfer parcellation did not allow us to test hypotheses about amygdala subdivisions, thus future work would benefit from investigating maltreatment-related neuroanatomical changes among distinct neural subdivisions in psychosis.

Despite the consistency of our CSA-amygdala findings with prior human and animal studies, these findings contrast with separate reports of lower amygdala volumes in association with maltreatment^56^ and with others showing no effect of maltreatment on amygdala volume^60^. Contrary to our expectations, we also found no evidence that CSA was associated with reduced GMV of the hippocampus. The impact of maltreatment on limbic structure volumes is complex and involves multiple modifying factors, including timing and chronicity of exposure^16^, stage of pubertal development^61^, genetic influences^62^, and protective factors such as social supports^63^. Notably, structure-specific patterns of neuronal remodeling following stress also likely influence the nature of maltreatment-related GMV changes: Whereas regions of the amygdala follow a pattern of dendritic hypertrophy following stress, the hippocampus and medial prefrontal cortex follow a pattern of stress-induced atrophy^19^. Interestingly, it has been proposed that the trajectory of maltreatment-related amygdala structural alterations may be nonlinear such that volume enlargement and sensitization after initial exposure is followed by volume reductions in response to subsequent or ongoing victimization^16^. Our cross-sectional study did not collect information regarding potential genetic, environmental, or developmental modifiers of CSA-amygdala associations; thus, an important future goal is to disentangle their potential effects on neurodevelopment and psychosis through longitudinal neuroimaging research. Future work will also benefit from the study of other stress-sensitive regions as they pertain to the link between maltreatment and psychotic disorders, such as the orbital and dorsolateral prefrontal cortices^17,18,60^.

Our finding of lateralized associations between CSA and amygdala volume also warrants discussion. Although we observed a positive and statistically significant association between CSA and amygdala volume in the left hemisphere (*r* = .30, *p* = .01), we also observed a positive but non-significant association between CSA and amygdala volume in the *right* hemisphere (*r* = .12, *p* = .33). Thus, it is possible that a qualitatively similar effect of CSA on amygdala volume was present within our sample bilaterally, but that we were only powered to detect significant findings in the left amygdala. It is also possible that CSA is associated with a reduction in normative volume asymmetry. Substantial evidence demonstrates that the left amygdala tends to be slightly smaller than the right amygdala in the healthy adult human brain^64,65^, and this asymmetry is often exaggerated among people with schizophrenia^49^. Importantly, our finding of lateralized associations between CSA and amygdala volume is consistent with research showing that stress can alter the formation of normative asymmetries of limbic and other structures^66^. As evidence indicates that the left (vs. right) amygdala is more specialized in supporting responses that are sustained^67,68^ or evoked by negative emotional material^68–70^, CSA-related amygdala reorganization could conceivably contribute to the heightened or prolonged stress responses common among individuals with psychosis^71^, which is seen particularly among those with AH^72^ or a history of maltreatment^73^. The significance of asymmetries in stress-sensitive brain regions and their intersection with maltreatment and psychotic disorder clearly merits further study.

This study should be considered in light of several limitations. First, our sample size was relatively modest, and we were not powered to examine differences in the relations of interest across AH and NAH within each diagnostic category (schizophrenia, schizoaffective disorder, and bipolar disorder). Although a strength of our study is the recruitment of a transdiagnostic sample and our results held when controlling for DSM diagnostic class, it is possible that CSA and/or CSA-AH relations present differently across these disorders. Similarly, factors such as race, culture, and gender identity represent important considerations in studies of maltreatment and putative clinical outcomes^74,75^, but our study was not positioned to address these factors given our modest sample size and lack of questions probing gender identity. Second, the AH group had considerably higher mean CPZ equivalents than the NAH group. Although our main findings held when including CPZ equivalents as a covariate in analyses, the potential contribution of medication load to our results cannot be fully ruled out. Third, our focus on GMV means we were unable to examine whether any CSA-associated amygdala alterations are active during emotion processing, learning, or other functions of the amygdala. Although examining neural trait correlates of psychosis is important, studies examining the effects of CSA on in vivo brain function in AH are also needed.

Fourth, our study was cross-sectional and thus inferences about the causal nature or timing of events (e.g., maltreatment, psychosis onset) should be made cautiously. Our measure of CSA was based on retrospective self-report, and evidence suggests significant disclosure delays and rates of nondisclosure among victimized individuals, particularly males^76–78^. Although several studies have demonstrated that self-reports of childhood trauma are highly reliable even in the presence of psychosis^79,80^ and we observed no evidence of differential reporting bias across sex or AH status, it remains possible that some childhood maltreatment exposures went undetected among patients or controls. Prospective longitudinal designs using multiple measures and/or sources of maltreatment information (e.g., family members, medical records, validated interviews^81^) may provide greater insights into how CSA and other forms of adversity may increase the risk of GMV changes and AH.

In summary, our findings are consistent with prior work suggesting that CSA is more common among individuals with AH vs. NAH. We extend these findings by showing this effect may be driven largely by the female sex, and also show an association between CSA and higher GMV in the left amygdala. Our findings highlight the importance of considering life experience and sex along with clinical presentation when formulating hypotheses about environmental and neurobiological contributors to psychosis.

## Methods

### Participants

This study was part of a larger neuroimaging study of AH in the Psychotic Disorders Division at McLean Hospital, a private psychiatric hospital affiliated with Harvard Medical School in Belmont, Massachusetts. Patients ages 18-50 were recruited from inpatient (*n* = 6) and outpatient (*n* = 114) services and included individuals with schizophrenia, schizoaffective disorder, or bipolar disorder with psychotic features. The effort was made to enroll a roughly equal number of participants with a history of AH within each diagnostic category (schizophrenia spectrum versus psychotic bipolar disorder). Healthy control participants with no current Axis I psychiatric disorder, no history of psychosis, and no first-degree relatives with psychotic disorders were recruited from advertisements in the community. Exclusion criteria for all groups included non-fluency in English, hearing impairment, clinically significant neurological or non-psychiatric medical conditions, MRI contraindications, electroconvulsive treatment within the past year, and DSM-IV-TR criteria for substance abuse in the prior three months or substance dependence in the prior five years. Most participants (80%) completed all procedures (clinical and neuroimaging) in a single visit. The study could be completed in more than one study visit if preferred by participants for comfort and convenience, or if rescanning was needed due to technical problems during the original scan; in such cases, all research procedures were completed within one month (mean time between clinical assessment and scan 1.7 days ± SD 4.6, range 0–27), with 94% of participants completing all procedures within a week. Data collection took place between November 2014 and March 2019. The study was approved by the Mass General Brigham Institutional Review Board, which oversees human subjects research at McLean Hospital. All participants provided written informed consent.

Primary diagnosis was determined using the Structured Clinical Interview for the DSM-IV-TR (SCID)^82^, administered by a trained research assistant under the supervision of a psychiatrist who specializes in psychotic disorders. Consistent with prior research^1,83^, lifetime AH was determined using the SCID item B16: “Did you ever hear things that other people couldn’t, such as noises, or the voices of people whispering or talking?” Patients scoring a 3 (threshold/true) were classified as AH and all other patients were classified as NAH. Assignments were made by a psychiatrist with expertise in psychosis. Current psychotic and affective symptom severity among patients was measured using standard assessments: the Psychotic Symptom Rating Scale’s auditory hallucinations subscale (PSYRATS-AH)^84^, the Scale for the Assessment of Positive Symptoms (SAPS)^85^, the Scale for the Assessment of Negative Symptoms (SANS)^86^, the Young Mania Rating Scale (YMRS)^87^, and the Montgomery-Asberg Depression Rating Scale (MADRS)^88^. In addition, participants completed the CTQ^89^, a 28-item self-report questionnaire that is widely used in psychopathology and trauma research. The measure captures experiences of CSA, physical abuse, emotional abuse, physical neglect, and emotional neglect, and includes a 3-item “minimization/denial” scale intended to signal evidence of underreporting. All items are rated on a 5-point Likert scale; minimization/denial positivity is identified when any of this scale’s 3 items (e.g., “I had the perfect childhood”) are endorsed at a level of 5. Cronbach’s alpha for the CTQ in the present sample was α = .86. Sex was determined by asking participants to report their sex (male or female; options for non-disclosure and more detailed questions regarding gender identity were added toward the end of data collection but were acquired in inadequate numbers for analysis).

### Image acquisition

We acquired high-resolution T1-weighted structural MRI data using a Siemens TIM Trio 3-Tesla MRI scanner and a 32-channel head coil at the McLean Imaging Center. We used a multi-echo magnetization prepared rapid acquisition gradient echo (ME-MPRAGE) sequence with TE1/TE2/TR 3.31/6.99/2530 ms, TI 1100 ms, flip angle 7°, FOV 256 mm, matrix size 256 ×256, 176 slices, 1.0mm^2^ in-plane resolution, and 1.0 mm slice thickness, with interleaved slices acquired along the anterior commissure-posterior commissure plane. The sequence utilized generalized auto-calibrating partially parallel acquisition (GRAPPA) with an acceleration factor of 2. The acquisition time for the T1-weighted image was 6:03 min.

### Image analysis

We used FreeSurfer 5.3 (http://surfer.nmr.mgh.harvard.edu/^90^) to perform automated segmentation of the T1-weighted data. This pipeline included: manual inspection for quality control of scans; generation of brain masks using multi-atlas brain segmentation^91^; removal of nonbrain tissue using a hybrid watershed/surface deformation procedure; automated Talairach transformation; segmentation of the subcortical white matter and deep gray matter structures; intensity normalization; delineation of the gray matter white matter boundary; automated topology correction; and surface deformation following intensity gradients to optimally identify the gray/white and gray/cerebrospinal fluid boundaries. Surface-based registration projected the Desikan parcellation to individual subjects^92^. For the present study, we a priori selected the amygdala and hippocampus as brain regions of interest, which studies have shown can be accurately measured using FreeSurfer^93^. We extracted the GMVs of these two regions in the left and right hemispheres, as well as the estimated total intracranial volume. To adjust for volume differences that may be due to inter-individual variations in head size, GMV was divided by intracranial volume.

### Statistical analyses

#### Preliminary analyses

We compared the AH, NAH, and control groups on demographic and clinical variables using chi-square tests and ANOVAs as appropriate, with post hoc *t-*tests for ANOVAs in the case of significant differences. For variables for which we planned parametric analyses, we examined histograms, frequency distributions, and normality estimates to determine whether they met assumptions of normality and homoscedasticity.

#### Primary analyses

Our three main questions were whether patients with AH show distinct CSA histories relative to NAH patients or healthy controls; whether this effect is moderated by sex; and whether the history of CSA is associated with limbic GMV. First, to obtain a simple picture of group differences in CSA, we computed a one-way ANOVA with the clinical group (AH, NAH, control) as the factor and CSA as the dependent variable. We then tested the hypothesis that any AH-NAH differences in CSA histories are accounted for by higher exposure to CSA among AH females by conducting an ANOVA, setting CSA scores as the dependent variable and clinical group, sex, and their interaction as factors. To evaluate the specificity and robustness of our findings, we followed up with one-way ANOVAs, setting AH status as the factor and each of the four non-CSA maltreatment domains from the CTQ as separate dependent variables. We also conducted an ANCOVA with AH status as the factor and sex, DSM diagnostic class (schizophrenia spectrum, bipolar disorder), CPZ equivalents, and non-CSA maltreatment dimensions as covariates. We examined rates of potential minimization/denial across sex and AH status by conducting a chi-square analysis with group (sex or AH status) and minimization/denial positivity as variables.

Second, to determine the magnitude of the potential relation between the current severity (versus lifetime experience) of AH, we computed a Pearson correlation between CTQ CSA scores and PSYRATS-AH scores. We then assessed the specificity of these findings by computing Pearson correlations between CSA scores and other symptom dimensions, including overall positive (SAPS), negative (SANS), manic (YMRS), and depressive (MADRS) symptoms. To ensure that our measure of overall positive symptoms was distinct from a measure of AH, we excluded the three AH items from the SAPS score. Similar to our analyses of group differences in CSA, in supplementary analyses we evaluated the robustness of the CSA-PSYRATS-AH correlation by computing two linear regression models: one in which PSYRATS-AH scores (analogous to AH status in the group difference analyses) were regressed on CSA scores, sex, and their interaction (to examine the moderating effect of sex on any CSA-symptom relation); and one in which PSYRATS-AH scores were regressed on CSA score, sex, DSM diagnostic class, and all other maltreatment dimensions (to examine the effects of covariates).

After establishing the relations between CSA and clinical variables as above, we sought to determine whether the severity of CSA exposure was related to GMV of the amygdala and hippocampus in each hemisphere in the combined patient group. To do this, we conducted Pearson correlations between CSA scores and regional GMV (adjusted for intracranial volume). In the case of significant findings, we planned two supplementary linear regressions: one in which GMV was regressed on CSA score, sex, and their interaction (to examine the moderating effect of sex on any CSA-GMV relation); and one in which GMV was regressed on CSA score, sex, AH status, DSM diagnostic class, CPZ equivalents, and all other maltreatment dimensions. The full patient sample was included to maximize statistical power, given the reduction in power introduced by including several critical covariates; AH status was added as a covariate to determine the effect of CSA on GMV for the average patient with AH or NAH.

## Supplementary information

Supplemental Material - Clean

## Data Availability

The data supporting the findings of this study are available from the corresponding author upon reasonable request.
