# Supplementary material for: Auditory hallucinations, childhood sexual abuse, and limbic gray matter volume in a transdiagnostic sample of people with psychosis
Source: Schizophrenia (Heidelb). 2022 Dec 30;8(1):118. doi: 10.1038/s41537-022-00323-y (PMC9803640; doi:10.1038/s41537-022-00323-y)
Supplement: Supplementary file 1 — Supplemental Material - Clean [file 41537_2022_323_MOESM1_ESM.docx]

**SUPPLEMENTARY MATERIAL**

**Table S1.** Analysis of covariance assessing differences in sexual abuse histories across healthy controls and patients with or without lifetime auditory hallucinations, adjusting for covariates.

| Variable | *F* | *p* |
| --- | --- | --- |
| AH status | 3.88 | .02 |
| Sex | 7.79 | .006 |
| DSM diagnostic class | 0.32 | .58 |
| CPZ equivalents | 1.06 | .31 |
| Physical abuse | 1.09 | .30 |
| Emotional abuse | 0.11 | .74 |
| Physical neglect | 2.76 | .10 |
| Emotional neglect | 0.07 | .80 |

Note: for AH status, *df* = 2, 106; for all other terms, *df* = 1, 106. DSM diagnostic class was coded as schizophrenia spectrum versus bipolar disorder. AH = lifetime auditory hallucinations, CPZ = chlorpromazine, DSM = diagnostic and statistical manual of mental disorders.

**Table S2.** Frequencies and proportions of participant minimization/denial scores plus chi square analyses assessing scale differences across sex and clinical group.

| Group | Positive Score on CTQ Minimization/ Denial Scale | | χ^2^ | *p* |
| --- | --- | --- | --- | --- |
|  | No | Yes |  |  |
|  |  |  |  |  |
| Male | 37 (66.1) | 19 (33.9) | 1.43 | .23 |
| Female | 42 (76.4) | 13 (23.6) |  |  |
|  |  |  |  |  |
| AH | 29 (72.5) | 11 (27.5) | 0.80 | .67 |
| NAH | 27 (75.0) | 9 (25) |  |  |
| Control | 23 (65.7) | 12 (34.3) |  |  |

Note: For sex comparison, *df* = 1; for clinical group comparison, *df* = 2. AH = lifetime auditory hallucinations, NAH = no lifetime auditory hallucinations.

**Table S3.** Linear regression model predicting current auditory hallucination severity from sexual abuse history, sex, and their interaction.

| Variable | *b (sb)* | *t* | *p* |
| --- | --- | --- | --- |
| Sexual abuse | 0.68 (0.34) | 1.99 | .05 |
| Sex | -9.12 (7.29) | -1.25 | .22 |
| Sexual abuse × sex | 2.24 (1.15) | 1.95 | .06 |

Note: *df* =72.

**Table S4**. Linear regression model predicting patients’ current auditory hallucination severity from sexual abuse history, adjusting for covariates.

| Variable | *b (sb)* | *t* | *p* |
| --- | --- | --- | --- |
| Sexual abuse | 0.77 (0.32) | 2.41 | .02 |
| Sex | 2.20 (2.92) | 0.75 | .45 |
| DSM diagnostic class | 4.30 (2.82) | 1.52 | .13 |
| CPZ equivalents | 0.01 (0.00) | 3.61 | .001 |
| Physical abuse | -0.46 (0.54) | -0.85 | .40 |
| Emotional abuse | 0.69 (0.58) | 1.19 | .24 |
| Physical neglect | 0.50 (0.60) | 0.84 | .41 |
| Emotional neglect | 0.23 (0.51) | 0.45 | .66 |

Note: *df* = 69. DSM diagnostic class was coded as schizophrenia spectrum versus bipolar disorder. CPZ = chlorpromazine, DSM = diagnostic and statistical manual of mental disorders.

**Table S5.** Linear regression model predicting left amygdala gray matter volume in patients from sexual abuse history, sex, and their interaction.

| Variable | *b (sb)* | *t* | *p* |
| --- | --- | --- | --- |
| Sexual abuse | 1.44 × 10^-5^ (0.00) | 2.17 | .03 |
| Sex | -1.38 × 10^-5^ (0.00) | -0.11 | .91 |
| Sexual abuse × sex | 2.41 × 10^-6^ (0.00) | 0.13 | .90 |

Note: *df* = 71.

**Table S6.** Linear regression model predicting left amygdala gray matter volume in patients from sexual abuse history, controlling for additional covariates.

| Variable | *b (sb)* | *t* | *p* |
| --- | --- | --- | --- |
| Sexual abuse | 1.69 × 10^-5^ (0.00) | 2.52 | .01 |
| Sex | 6.64 × 10^-6^ (0.00) | 0.13 | .90 |
| AH status | 3.98 × 10^-5^ (0.00) | 0.81 | .44 |
| DSM diagnostic class | -1.34 × 10^-5^ (0.00) | -0.26 | .79 |
| CPZ equivalents | -3.38 × 10^-8^ (0.00) | -0.40 | .69 |
| Physical abuse | -1.97 × 10^-6^ (0.00) | -0.21 | .84 |
| Emotional abuse | 4.09 × 10^-6^ (0.00) | 0.41 | .68 |
| Physical neglect | -2.12 × 10^-5^ (0.00) | -2.04 | .05 |
| Emotional neglect | -1.21 × 10^-6^ (0.00) | 0.13 | .89 |

Note: *df* = 67. DSM diagnostic class was coded as schizophrenia spectrum versus bipolar disorder. AH = lifetime auditory hallucinations, DSM = diagnostic and statistical manual of mental disorders.
